# Supplementary material for: Identification of photoperiod-induced specific miRNAs in the adrenal glands of Sunite sheep (Ovis aries)
Source: Front Vet Sci. 2022 Jul 22;9:888207. doi: 10.3389/fvets.2022.888207 (PMC9354845; doi:10.3389/fvets.2022.888207)
Supplement: Supplementary file 1 [file Data_Sheet_1.ZIP › Supplementary file/miRDeep2 file.zip!/miRDeep2 file/Novel_Structure/LP42A3/Novel_212.pdf]

Provisional ID	:	NC_019480.2_22244
Score total	:	2.8
Score for star read(s)	:	3.9
Score for read counts	:	-3.3
Score for mfe	:	0.7
Score for randfold	:	1.6
Score for cons. seed	:	
Total read count	:	5
Mature read count	:	4
Loop read count	:	0
Star read count	:	1

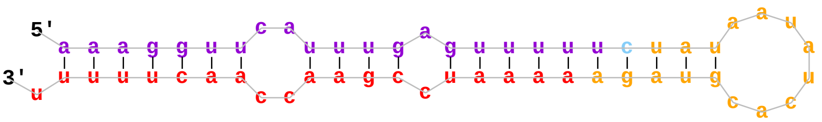

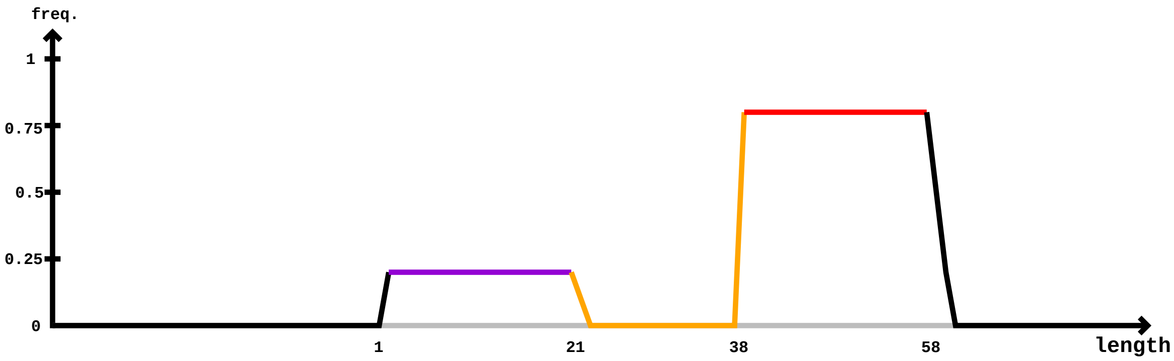


```
                                 Star                                 Mature
5'- uauuucuuccucaacauuauauuggguuagccaaaagguucauuugaguuuuucuauaauaucacguagaaaaauccgaaccaacuuuuuggucaagccaagaauugguaua   -3'    obs
    uauuucuuccucaacauuauauuggguuagccaaaagguucauuugaguuuuucuauaauaucacguagaaaaauccgaaccaacuuuuuggucaagccaagaauugguaua          exp
    .............((......((((.((.(((((((((((..((((.((((((((((........)))))))))).))))..))))))))))).)).))))......))...   reads   mm   sample
    .................................aaagguucauuugGguuuuu...........................................................   1       1    seq
    ......................................................................aaaauccgaacAaacuuuuu......................   3       1    seq
    ......................................................................aaaauccgaaccaacuuuuugg....................   1       0    seq
```
